# Characterization of polymorphic microsatellite markers for the Japanese endangered land snail *Mandarina*

**DOI:** 10.1186/s13104-022-06147-4

**Published:** 2022-07-16

**Authors:** Shu Nishida, Kotaro Mimura, Hideaki Mori, Satoshi Chiba

**Affiliations:** 1 Graduate School of Life Sciences, Tohoku University, 6-3 Aramaki aza aoba, Aoba-ku, Sendai, Miyagi 980-8578 Japan; 2 Japan Wildlife Research Center, Kotobashi 3-3-7 Sumida-ku Tokyo, Tokyo, 130-8606 Japan; 3 Center for Northeast Asian Studies, Tohoku University, 12-2, Kawauchi, Aoba-ku, Sendai, Miyagi 980-8578 Japan

**Keywords:** Nuclear microsatellite, *Mandarina*, *Mandarina mandarina*, Endangered species, Oceanic land snails, Ogasawara islands

## Abstract

**Objective:**

*Mandarina* is an endangered land snail genus of the oceanic Ogasawara archipelago. On Chichijima Island, the largest inhabited island in Ogasawara, this genus is almost extinct in the wild due to predation by invasive species. Although ex situ conservation programs started in 2010, genetic diversity and population structure remain unclear due to a lack of genetic markers with sufficient genetic variation. In this study, we designed polymorphic microsatellite markers of *Mandarina* to enable genetic analysis and to develop appropriate conservation plans.

**Results:**

Twenty-three polymorphic microsatellite markers were identified from the genomic DNA of wild samples of *Mandarina mandarina*. We assessed the genetic diversity of each marker. In 16 markers, neither linkage disequilibrium nor deviation from Hardy–Weinberg equilibrium was detected. These 16 markers were tested for multiplex PCR using low-density DNA extracted non-lethally from captive samples of *M. mandarina*, *M. chichijimana* and *M. suenoae*. Of the 16 markers, 15, 12 and 9 were usable for multiplex PCR, respectively. Genetic analysis using these microsatellite loci will be an important resource for the conservation of *Mandarina*.

**Supplementary Information:**

The online version contains supplementary material available at 10.1186/s13104-022-06147-4.

## Introduction

*Mandarina* is a Japanese endemic genus of land snails that is highly diversified in the oceanic Ogasawara archipelago [1, 2]. This genus symbolizes the Ogasawara Islands’ value as a World Natural Heritage site, but its population has declined seriously due to the impact of invasive species [3]. In particular, *Mandarina* species on Chichijima Island, the largest inhabited island in Ogasawara, are near extinction in the wild due to predation by the invasive malacophilous flatworm *Platydemus manokwari* [2]. Ex situ conservation programs for the *Mandarina* of Chichijima Island were initiated in 2010 [4].

Genetic analysis is an essential part of ex situ conservation. Bottlenecks at the start of captive breeding and inbreeding in captivity can alter the genetic structure and genetic diversity of populations [5–8]. Therefore, genetic analysis should be conducted in parallel with captive breeding, but genetic markers with sufficient intraspecific variation have not been developed for *Mandarina* on Chichijima Island. Although microsatellite markers were used for population genetic analysis of *Mandarina* species on Hahajima Island [9, 10], these markers did not work for the species on Chichijima Island. In the ex situ conservation of the Hawaiian *Achatinella lila*, also an oceanic land snail, the population began to decline, likely due to bottlenecks, approximately 10 years after the start of reproduction [7]. In *Mandarina*, 12 years after the start of the artificial breeding program, the lack of genetic analysis is an issue that must be addressed urgently.

Microsatellite markers are suitable for genetic profiling in the conservation of non-model organisms. Their advantages include a fast evolutionary rate, high intraspecific variation, low-cost experimental methods using multiplex PCR, and the possibility of diverting markers among related species [11, 12]. In addition, the ability to analyze tiny amounts of DNA collected in a non-lethal manner allows for genetic analysis in parallel with captive breeding [7]. Genetic analysis using microsatellite markers is expected to make a significant contribution to the development of breeding plans that avoid risks such as genetic degradation and changes in population structure. In this study, we developed polymorphic microsatellite markers for *Mandarina mandarina* and evaluated their potential use in the related species, *M. chichijimana* and *M. suenoae*. In addition, we examined the feasibility of using these markers in multiplex PCR using tiny amounts of DNA as a non-lethal, low-cost experimental method that is expected in conservation settings.

## Main text

### Materials and methods

DNA was extracted from the muscle tissues from the foot of *M. mandarina* using the NucleoSpin Tissue kit (TaKaRa). Microsatellite sequences were isolated by Ecogenics GmbH (Switzerland). Size-selected fragments from the genomic DNA of *M. mandarina* were enriched for simple sequence repeat (SSR) content using magnetic streptavidin beads and biotin-labelled GATA and GTAT repeat oligonucleotides (Ecogenics GmbH). The SSR-enriched library was analyzed on the Illumina MiSeq platform using the Nano 2 × 250 v2 format (Ecogenics GmbH). Microsatellite loci were selected if the number of repeat motifs was 2–4 and if the number of repeats was greater than 10. In total, 54 primer pairs were developed (Additional file 1) and tested for amplification and polymorphism using 24 wild samples of *M. mandarina* collected from Chichijima island (Additional file 2). Fluorescent-labelled universal primers were added to the forward primers for fluorescent dye labelling [13]. PIG-tails were attached to the reverse primers to reduce adenylation [14]. Three primer sequences, namely FAM-tail (5ʹ-FAM-GCCTCCCTCGCGCCA-3ʹ), VIC-tail (5ʹ-VIC-GCCTTGCCAGCCCGC-3ʹ), NED-tail (5ʹ-NED-CAGGACCAGGCTACCGTG-3ʹ) [15], were employed as universal primers. PCR amplifications were performed for single primer sets. The PCR solutions were 2 µL and contained the following components: 0.1–5 ng of genomic DNA dried at the bottom of the reaction tube in advance, 1.2 µL of Type-it Multiplex PCR Master Mix (QIAGEN), 0.03 µM tailed forward primer, 0.13 µM fluorescent-labelled universal primer and 0.13 µM reverse primer. The reactions had an initial denaturation step at 95 °C for 15 min, followed by 40 cycles at 94 °C for 30 s, 60 °C for 90 s, 72 °C for 60 s and finally 60 °C for 30 min. Product sizes were determined using the ABI 3130xl Genetic Analyzer and Peak Scanner software (Applied Biosystems) with GeneScan 500 LIZ dye Size Standard v2.0 (Applied Biosystems).

Genetic variability was calculated using GenALEx 6.5 [16]. Linkage disequilibrium and deviations from Hardy–Weinberg equilibrium (HWE) were calculated using Genepop Web version 4.2 [17]. Probabilities of individual identity (PID) and probabilities of sibling individual identity (PID [sib]) were estimated using CERVUS v3.0.7 [18]. For each microsatellite loci and cumulative PID and PID (sib) were calculated by multiplying PID or PID (sib) value across all loci. The multi-locus PID was calculated by multiplying PID for each locus.

Polymorphic microsatellite loci that showed neither linkage disequilibrium nor deviation from HWE were tested for multiplex PCR using captive samples of *M. mandarina* (N = 34), *M. chichijimana* (N = 69), and *M. suenoae* (N = 19) (Additional file 2). *M. chichijimana* is a sister species of *M. mandarina*, while *M. suenoae* is a more distantly related species [2]. DNA was extracted from the muscle tissues approximately 2 mm from the tip of the foot. This method can be used to collect DNA from captive individuals non-lethally, but the quantity of extracted DNA is low. Multiplex PCR was performed by simultaneously amplifying 2–4 sets of markers labelled with different fluorescent colours. (Additional file 3). The PCR solutions were 2 µL and contained the following components: 0.01–5 ng of genomic DNA dried at the bottom of the reaction tube in advance, 1.0 µL of Type-it Multiplex PCR Master Mix (QIAGEN), 0.2 µM fluorescent-labelled forward primer and 0.2 µM reverse primer for each primer sets. The reactions had an initial denaturation step at 95 °C for 15 min, followed by 35 cycles at 94 °C for 30 s, 60 °C for 90 s, 72 °C for 60 s and finally 60 °C for 30 min. Product sizes were determined using an ABI 3130xl Genetic Analyzer and Peak Scanner software (Applied Biosystems) with GeneScan 500 LIZ dye Size Standard v2.0 (Applied Biosystems).

### Results

Twenty-three polymorphic microsatellite loci were identified from wild sample of *M. mandarina* (Table 1). Six to twenty alleles (average = 13.9) were detected; observed heterozygosity were 0.33–0.78 (average = 0.61), while expected heterozygosity showed higher value range of 0.50–0.92 (average = 0.87). PID ranged from 0.011 to 0.060, and PID(sib) ranged from 0.29 to 0.57. Multi-locus PID values, when using eight or more were sufficiently low to distinguish individuals or siblings (P < 0.0001 [19]) (Table 1). Linkage disequilibrium was not detected in any marker, while seven loci showed deviation from HWE (P < 0.05). We tested the remaining 16 loci for multiplex PCR using captive samples.

**Table 1:** Characterization of the polymorphic microsatellite markers for *Mandarina mandarina*

Locus	Primer sequence (5'–3')	Repeat motif	Size range (bp)	Na	Ho	He	Fis	Pid	Pid (sib)
Manman_12260B	F: AGCGTTACATACGTCACTTTGG R: ACGAATAGTGGTGTCTACCCG	CTG	230–287	15	0.708	0.906	0.218	0.016	0.30
Manman_161603	F: TGCGGTCTTATTTCATTGCAG R: CAACAGATAGGCTTGCCGTG	TTC	168–189	8	0.625	0.837	0.253	0.047	0.34
Manman_247740	F: TCATAGTGACTGCACATGCC R: TGTTCAAACGATTTCCAAAAAGC	TAA	227–269	13	0.522	0.889	0.413	0.023	0.31
Manman_392524	F: GGTTTAAGACGATGGCACGG R: TTAATTCTTTAACAACGGCAAGC	ATG	249–279	10	0.696	0.846	0.178	0.042	0.34
Manman_378362	F: TCAGAACTATTCCTGTTGTGTGC R: ATTAAACTGCAAGCGGTTCG	TC	174–206	14	0.708	0.896	0.209	0.019	0.31
Manman_6672	F: GAGCAGTATCATCCCCTTGC R: CACGCCCAGAAAATAAAAACTATCG	TCA	242–278	13	0.783	0.894	0.125	0.020	0.31
Manman_31975	F: CGAGGCATCTCCAAAAACAC R: TTATGGGTCTGCCCCGAG	TGA	105–123	6	0.375	0.498	0.247	0.380	0.57
Manman_308475	F: TCATGACCTCTGTCCTATGCC R:TCTCGAGTTGACTGTTTAGTATCATC	ATG	154–193	13	0.583	0.885	0.341	0.239	0.31
Manman_1709	F: ATAGAATGGGGTCGTGCCTC R: TCGACGCTGACAAGCAAATG	TTC	230–317	20	0.792	0.927	0.151	0.011	0.29
Manman_7787A	F: GATATGCTGTTGGAGGTCGC R: TGTCTGTTAATTGCCCACGC	TGA	257–326	16	0.583	0.876	0.381	0.025	0.32
Manman_30938	F: GGCTTTGTTTTGTAGCACCG R: ACTTTGTGATCTCCCAGTCG	GTTA	227–279	14	0.625	0.885	0.293	0.024	0.31
Manman_118744	F: ACTATGACGCACCATCTCCC R: GAGTCAACTTGCCCATCACAG	GAT	118–148	11	0.667	0.799	0.165	0.060	0.37
Manman_235472	F: GGTTCTATGGGGTTTACAGCG R: TCCATATACTGACATTGTTTGCC	TAT	267–336	15	0.625	0.908	0.312	0.016	0.30
Manman_46250	F: CATGGCGAACCCTAATCTGC R: TGGGTCTGACTATATGAGGGC	AG	175–207	14	0.667	0.871	0.234	0.028	0.32
Manman_82352B	F: AGAACAACGGGGTACTGGAG R: GCTTGCGTGTCCGATTTTTAAC	GA	223–285	17	0.708	0.905	0.217	0.016	0.30
Manman_4034†	F: CGCTTCGGTCTAGAATGCAC R: ACTCCATCTTAGCCTCTGTCG	AG	221–261	15	0.750	0.917	0.182	0.013	0.30
Manman_209567*	F: AGGCATGTTTTCTTTTTAGATAATGG R: TATTGTGCACTGAGCTCGAC	GA	263–299	14	0.542	0.879	0.384	0.026	0.32
Manman_345444*	F: ATCGTACCAGACATCTGCCC R: GGCTGTCAGGGCCATTATTC	GA	160–206	16	0.696	0.878	0.208	0.023	0.32
Manman_13329A*	F: TGTACACGAGCTTTTCGACC R: CGATGCACGAATTGAGTCCC	GA	128–152	11	0.500	0.865	0.422	0.032	0.33
Manman_140288*	F: CGCGTTCCCCAATACTCAAC R: ACAGTGTCTGAAACCGGTAG	TTC	204–276	20	0.625	0.919	0.320	0.012	0.29
Manman_183849*	F: GGCTACGGCATAAGGTGAG R: GGAATACATCCTGCAAGCGG	GAT	168–207	11	0.333	0.870	0.617	0.031	0.32
Manman_82799*	F: CACCAGGAGTCGCAAATGAC R: ACTGCCATCAGGTTGTGAAG	AG	121–183	18	0.458	0.923	0.503	0.011	0.29
Manman_90631*	F: TAGATGTGGTGGAGGCATCG R: CCCTTAGCGTATCTCCACAGG	GA	242–302	16	0.478	0.907	0.473	0.016	0.30

*Na* the number of alleles, *Ho* observed heterozygosity, *He* expected heterozygosity, *Fis* inbreeding coefficient. *PID* probability of identity, *PID (sib)* probability of siblings

A dagger ^†^indicates that loci were not clearly amplified in multiplex PCR

An asterisk ^*^indicates that loci showed deviation from hardy–Weinberg equilibrium (P < 0.05)

In all of the species, multiple loci were identified for which multiplex PCR with low-density DNA was possible. Among captive samples of *M. mandarina*, one of the 16 loci, Manman_4034, did not show distinct amplification, probably due to interference between primers in multiplex PCR or a low density of DNA. In the other 15 loci, the average number of alleles was 12.2, and the average observed and expected heterozygosity values were 0.63 and 0.83, respectively (Table 2). In the case of *M. chichijimana*, a sister species, 12 loci showed distinct amplification and polymorphism. In these 12 loci, the average number of alleles was 17.8, and the average observed and expected heterozygosity values were 0.68 and 0.88, respectively (Table 2). Among the samples of *M. suenoae*, a more distant species from *M. mandarin*a, nine loci showed distinct amplification and polymorphism. Of these nine loci, the average number of alleles was 6.7, and the observed and expected heterozygosity values were both 0.75 (Table 2).

**Table 2:** Genetic diversity of captive samples of *M. manadrina*, *M. chichijimana* and *M. suenoae*

	*M. mandarina* (N = 34)			*M.chichijimana* (N = 69)			*M.suenoae* (N = 19)		
	Na	Ho	He	Na	Ho	He	Na	Ho	He
Manman_12260B	13.0	0.38	0.87	–	–	–	–	–	–
Manman_161603	9.0	0.56	0.78	10.0	0.59	0.80	6.0	0.90	0.80
Manman_247740	16.0	0.71	0.91	13.0	0.70	0.88	–	–	–
Manman_392524	8.0	0.56	0.80	–	–	–	7.0	0.90	0.78
Manman_378362	11.0	0.88	0.85	16.0	0.67	0.85	7.0	0.90	0.78
Manman_6672	12.0	0.65	0.87	13.0	0.67	0.85	5.0	0.58	0.61
Manman_31975	7.0	0.71	0.60	9.0	0.58	0.73	–	–	–
Manman_308475	13.0	0.68	0.85	18.0	0.63	0.92	–	–	–
Manman_1709	15.0	0.68	0.91	32.0	0.64	0.95	11.0	0.63	0.87
Manman_7787A	14.0	0.61	0.82	–	–	–	6.0	0.79	0.76
Manman_30938	12.0	0.71	0.84	13.0	0.81	0.88	6.0	0.58	0.66
Manman_118744	7.0	0.38	0.68	18.0	0.58	0.91	–	–	–
Manman_235472	13.0	0.59	0.89	27.0	0.83	0.93	5.0	0.74	0.73
Manman_46250	17.0	0.73	0.91	24.0	0.74	0.92	7.0	0.74	0.72
Manman_82352B	16.0	0.71	0.89	21.0	0.71	0.90	–	–	–
Average	12.2	0.63	0.83	17.8	0.68	0.88	6.7	0.75	0.75

*Na* the number of alleles, *Ho* observed heterozygosity, *He* expected heterozygosity

### Discussion

The large number of alleles in the 23 loci (an average of 13.3 for 24 wild samples) was consistent with a previous study on *Mandarina* on Hahajima Island [10]. *Mandarina* is estimated to have evolved rapidly in Ogasawara, and high allele diversity may be related to this history. The number of markers available in common corresponded to the phylogenetic relationships among the three species, with more loci available for *M. chichijimana*, which is closely related to *M. mandarina*. In addition to the three species in this study, *M. hirasei* and *M. tomiyamai*—distantly related species of *M. mandarina*—are begging benefit from breeding programs. Further testing is needed to determine whether the respective markers can be used in these related species.

We generated polymorphic microsatellite markers that will be useful for the conservation of *Mandarina*. These markers will contribute to the clarification of the unknown population genetic structure of *Mandarina* and will help in the establishment of conservation units. Moreover, these markers were available for multiplex PCR using non-lethally obtained DNA. This method allows low-cost analysis of changes in genetic diversity in parallel with breeding, and it is expected to contribute to the development of appropriate breeding plans. Furthermore, the gene flow and population dynamics of *Mandarina* revealed by microsatellite analysis will be an important resource for elucidating the adaptive radiation process of these land snails on oceanic islands.

### Limitation

We did not test the optimal primer set combination for multiplex PCR. Some combinations may increase or decrease the amplification quality of microsatellite loci.

It is possible that deviations from HWE were detected at unproblematic markers because of the small number of wild samples compared to allelic diversity. Finally, these microsatellite markers have not been tested for species from islands other than Chichijima.

## Supplementary Information

**Additional file 1. **List of microsatellite markers tested in this study. The table includes locus name, repeat motif, amplicon size, forward and reverse primer sequences, and fluorescent label. A dagger (‡) indicates that loci were monomorphic. An asterisk (*) indicates that loci showed unclear or no amplification.

**Additional file 2. **List of samples used in this study. The table includes sample name, species name, wild or captive, sampling year (or birth year in the case of captive samples), and sampling site (or founders’ sampling site in the case of captive samples).

**Additional file 3. **List of combinations of markers in the multiplex PCR. The table includes combination name, loci in each combination, and fluorescent label. A dagger (‡) and an asterisk (*) indicate that loci were not available for *M. chichijimana* and *M. suenoae*, respectively.

## Data Availability

The datasets generated and/or analyzed during the current study are not publicly due to their use in ongoing publication but are available from the corresponding author on reasonable request.

## Abbreviations

bp: Base pair
Na: Number of alleles
Ho: Observed heterozygosity
He: Expected heterozygosity
Fis: Inbreeding coefficient
HWE: Hardy–Weinberg equilibrium
PID: Probabilities of individual identity
PID (sib): Probabilities of sibling individual identity
PCR: Polymerase chain reaction
